# 5-Year health-related quality of life outcome in patients with idiopathic normal pressure hydrocephalus

**DOI:** 10.1007/s00415-021-10477-x

**Published:** 2021-03-02

**Authors:** A. Junkkari, H. Sintonen, N. Danner, H. K. Jyrkkänen, T. Rauramaa, A. J. Luikku, A. M. Koivisto, R. P. Roine, H. Viinamäki, H. Soininen, J. E. Jääskeläinen, V. Leinonen

**Affiliations:** 1 Neurosurgery of NeuroCenter, Kuopio University Hospital and University of Eastern Finland, 70029 KYS, POB 100, Kuopio, Finland; 2 Department of Public Health, University of Helsinki, Helsinki, Finland; 3 Department of Pathology, Kuopio University Hospital and University of Eastern Finland, Kuopio, Finland; 4 Department of Neurology, University of Eastern Finland, Kuopio, Finland; 5 Neurology of NeuroCenter, Kuopio University Hospital, Kuopio, Finland; 6 Department Neurology, University of Helsinki, Helsinki, Finland; 7 Department Neurology, Helsinki University Hospital, Helsinki, Finland; 8 Department of Health and Social Management, University of Eastern Finland, Kuopio, Finland; 9 Department of Psychiatry, Kuopio University Hospital and University of Eastern Finland, Kuopio, Finland

**Keywords:** Health-related quality of life, 15D, Idiopathic normal pressure hydrocephalus, Charlson Age Comorbidity Index, Frontal cortical biopsy, Comorbidity

## Abstract

**Background:**

Health-related quality of life (HRQoL) is severely impaired in persons with idiopathic normal pressure hydrocephalus (iNPH). The HRQoL improves in a number of patients after the placement of a cerebrospinal fluid (CSF) shunt, but long-term follow-up of HRQoL is rare.

**Methods:**

Extended follow-up (60 months) of a prospective cohort study involving 189 patients with iNPH who underwent shunt surgery. Preoperative variables were used to predict favorable HRQoL outcome (improvement or non-deterioration) measured by the 15D instrument 5 years after shunting.

**Results:**

Out of the 189 initially enrolled study participants, 88 had completed 5-year HRQoL follow-up (46%), 64 had died (34%), and 37 (20%) failed to complete the HRQoL follow-up but were alive at the end of the study. After initial post-operative HRQoL improvement, HRQoL deteriorated so that 37/88 participants (42%) had a favorable HRQoL outcome 5 years after shunting. Multivariate binary logistic regression analysis indicated that younger age (adjusted OR 0.86, 95% CI 0.77–0.95; *p* < 0.005), lower body mass index (adjusted OR 0.87, 95% CI 0.77–0.98; *p* < 0.05) and better Mini-Mental State Examination performance (adjusted OR 1.16, 95% CI 1.01–1.32; *p* < 0.05) before surgery predicted favorable 5-year outcome.

**Conclusions:**

This extended follow-up showed that the self-evaluated HRQoL outcome is associated with iNPH patients’ pre-operative cognitive status, overweight and age. The post-operative deterioration may reflect the natural progression of iNPH, but also derive from aging and comorbidities. It indicates a need for long-term follow-up.

**Supplementary Information:**

The online version contains supplementary material available at 10.1007/s00415-021-10477-x.

## Introduction

Seven studies have evaluated health-related quality of life (HRQoL) in patients with idiopathic normal pressure hydrocephalus (iNPH) prior to and after cerebrospinal fluid (CSF) shunting (Table 1) [1–7]. A significant heterogeneity exists among the published studies: six different HRQoL instruments were applied, follow-up times ranged from 3 to 45 months and there was a lack of studies replicating and confirming previous findings (Table 1) [1–7]. Factors associated with poorer HRQoL outcome in iNPH in uni- or multivariate analyses have been identified: depressive symptoms [2], larger comorbidity burden [2, 4, 6], obesity [4], hyperlipidemia [2], severity of gait impairment [2], post-operative complications [5] and beta amyloid (Aβ) or hyperphosphorylated tau (HPτ) pathology in the frontal cortical biopsy [4]. While five out of seven (71%) studies observed significant HRQoL improvement following CSF shunting, only three studies reported HRQoL improvement rates [2, 4, 5], that ranged from 43 to 83% (Table 1) [1–7]. These three studies were also the only ones having controls from the general population, two of which compared HRQoL between their cohort and controls [2, 5]. When it comes to improvement of limited dimensions of HRQoL, gender (female) has been associated with greater improvement in mental health [6] and urinary symptoms [3] following surgery. While persons with iNPH having post-operative complications are justifiably thought to report lower HRQoL [4–6, 8], only one study has shown difference in HRQoL to those without any complications [5]. Here we aim to report and predict long-term HRQoL outcome in patients with iNPH.

**Table 1:** Results of the literature search

Search words used	(quality of life) and (normal pressure hydrocephalus)
Articles found from MEDLINE	62
Articles included^a^	7

Author(s) year	Country	Study type	Number of patients	HRQoL instrument	HRQoL follow-up time	HRQoL outcome	Dimensions of HRQoL that improved	Predictors of the quality of life outcome	Control population
Tsimiklis et al. (2020) [1]	Australia	Prospective cohort	20	AQoL-6D	6 months	Statistically significant improvement, outcome ratio cannot be determined	Cannot be determined		
Israelsson et al. (2020) [2]	Sweden	Retrospective cohort	176	EQ-5D-5L	Mean 21 months (6–45 months)	Improved in 132 (75%) of patients	Mobility, self-care, usual activity, pain/discomfort, anxiety/depression	Depressive symptoms, severity of gait disturbance, hyperlipidemia, high number of comorbidities	368 age- and gender-matched controls
Krzastek et al. (2017) [3]	USA	Prospective cohort	23	ICIq-LUTqol	Mean 10 months (3–24 months)	Statistically significant improvement, outcome ratio cannot be determined	Physical activities, urinary symptoms	In certain HRQoL dimensions: gender (female)	
Junkkari et al. (2017) [4]	Finland	Prospective cohort	145	15D instrument	12 months	improved in 63 (43%) of patients	Mobility, excretion	Body mass index, frontal cortical biopsy	Age-and gender-standardized sample of 3374 from general population were used as reference
Petersen et al. (2014) [5]	Sweden	Prospective cohort	37	EQ-5D-3 L	6 months	Improved in 31 (86%) of patients	Mobility, self-care, usual activities of daily living, anxiety/depression	Post-operative complications	Age matched sample of 1167 general population
Meier et al. (2013) [6]	Germany	Randomized trial	143	SF-12	12 months	Statistically significant improvement, outcome ratio cannot be determined	Physical composite score, mental composite score	Age adjusted Charlson comorbidity index score In certain HRQoL dimensions: Gender (female)	
Katzen et al. (2011) [7]	USA	Prospective cohort	12	SF-12	6 months	No statistically significant improvement, outcome ratio cannot be determined			

^a^The inclusion criteria were: (1) persons with idiopathic normal pressure hydrocephalus (not grouped with other conditions), (2) standardized quality-of-life questionnaire and (ii) follow-up after surgery, (3) not a review

*AQoL-6D* The Assessment of Quality of Life 6D questionnaire, *EQ-5D-5L* The 5-level EQ-5D version, *EQ-5D-3 L* The 3-level version of EQ-5D, *ICIq-LUTSqol* International Consultation on Incontinence Questionnaire Lower Urinary Tract Symptoms Quality of Life Module, *SF-12* 12-Item Short Form Survey

## Methods

This paper presents the results of an extended follow-up (60 months) of a prospective cohort study involving 189 patients with probable or possible iNPH [4]. A detailed description of the cohort population, methodology and results at 12 months have already been reported [4]. Here we present a brief summary of methods from the original cohort followed by a detailed description of the extended follow-up.

## Brief summary of methods used in original cohort study

### Design, participants and setting

The study was conducted in Kuopio University Hospital, a tertiary hospital center, which geographically provides all intracranial neurosurgery approximately to the 815,000 inhabitants of Eastern Finland. The KUH protocol for pre-operative workup and patient selection for shunt surgery and the characteristics of this cohort have been described in detail previously [4, 9].

### Data collection and outcome indicators

Primary outcome: HRQoL outcome 60 months postoperatively. As in the original study, HRQoL was evaluated using the 15D instrument at baseline, 3, 12 [4] and 60 months postoperatively. The HRQoL questionnaires were completed by the patients themselves or by an interviewing registered nurse. Secondary outcomes included reduction in iNPH -related symptoms measured by the 12-point iNPH grading scale (INPHGS), complications and survival.

### Health-related quality of life

The 15D is a generic HRQoL instrument with both profile and single index score properties [10]. The single index score (15D score) expresses the overall HRQoL on a 0–1 scale (1 = perfect health, 0 = dead) [10]. The dimension level values reflect the goodness of the levels relative to no problems on the dimension (= 1) and to being dead (= 0) [10]. Both are calculated from the health state descriptive system (questionnaire) using a set of population-based preference or utility weights [10]. A minimum clinically important change/difference in the 15D score has been estimated to be ± 0.015 [11].

For this study, we specified the definition of a favorable HRQoL outcome for iNPH patients. In the previous study [4], only iNPH patients who had experienced at least a minimum clinically significant improvement in HRQoL were considered to have favorable HRQoL outcome. To take into account the potential progression of the disease, iNPH patients who experience at least a minimum clinically significant improvement in HRQoL or their HRQoL remains the same (∆15D score > − 0.015) are considered in this study to have a favorable iNPH HRQoL outcome.

### Controls

To investigate in which degree age and aging affect the HRQoL, the 15D score and profiles of two age- and gender-standardized samples from the general population were used as references [12]: a population sample of 3372 persons at baseline and a population sample of 2906 persons at 5 years after shunting. The difference in these sample sizes is due to the difference in the age and gender composition of the patient group in these two points of time [12].

### Evaluation of iNPH symptoms

To classify the triad of symptoms we used a modified Finnish version of the 12-point iNPH grading scale (iNPHGS) [13]. The iNPHGS is a clinician-rated scale to separately assess the severity of each of the three core symptoms, with scoring based on observations by the physician and interviews with the patients or their caregivers [13].

### Cognitive evaluation

Mini-Mental State Examination (MMSE, range 0–30) was used to evaluate patients’ cognitive function [14]. As the primary education in Finland lasts for 9 years, patients were dichotomized according to years of education: patients with ≤ 9 years of education, and patients with > 9 years of education.

### Evaluation of depressive symptoms

Depressive symptoms were assessed with the self-administered 21-item Finnish version of the Beck Depression Inventory (BDI) [15] in a subpopulation of this cohort4.

### Comorbidities

The burden caused by the comorbidities was evaluated using the age adjusted Charlson Age Comorbidity Index (ACCI) [16].

### Biopsy procedure and immunohistochemistry

The details of the biopsy procedure and immunohistochemistry analysis in this cohort have been previously described in detail [4]. The cellular or neuritic immunoreactivity for Aβ and HPτ were evaluated by light microscopy in all samples and were graded as present or absent by a neuropathologist [17]. For statistical analyses, the patients were then further categorized by the presence of pathology of any kind: Aβ or HPτ found in the frontal cortical biopsy.

### Causes of death

The death certificates were obtained from the database of Statistics Finland. Causes of death were classified using ICD-10 diagnosis codes (International Statistical Classification of Diseases and Related Health Problems 10th revision) and were grouped according to a larger iNPH survival study [18].

### Statistical analysis

The data were analyzed using the Statistical Package for Social Sciences (SPSS^®^ 25 for Windows) and R language and environment for statistical computing (R-3.5.3 for Windows; R Development Core Team, R Foundation for Statistical computing, Vienna, Austria). The primary outcome variable was the HRQoL (15D). The paired samples *t* test or the Wilcoxon Sign test were applied to test differences in the means or the ranks of the repeated measurements in multiple comparisons, respectively. Independent samples *t* test was used to compare the mean 15D scores between the cohort and the general population.

The multivariate binary logistic regression analysis was performed using the enter method. Multivariate Cox Proportional-Hazards Model was conducted for mortality analysis. The odds ratios (ORs) and hazard ratios (HZs) were calculated with 95% confidence intervals (CIs). All tests for significance were two-sided, with probabilities of < 0.05 accepted as statistically significant. The Cox Proportional-Hazards Model was conducted for mortality analysis. The variable selection of statistical models was based on clinical significance and previously established predictors (Table 1) [1–7]. Due to drop-out/missing data, additional steps to detect signs of attrition bias were taken: when all variables (including outcome indicators) were analyzed at the same time to detect systematic tendencies in missing data, no significant tendencies were observed (Little’s Missing Completely at Random-test; *p* = 0.067). Additional logistic regression models were conducted, where we tried to predict the patient to be in the subgroup of 37/189 patients that were alive but did not complete the study (with both pre-and post-operative variables). No significant predictors were observed.

## Results

**Fig. 1:**
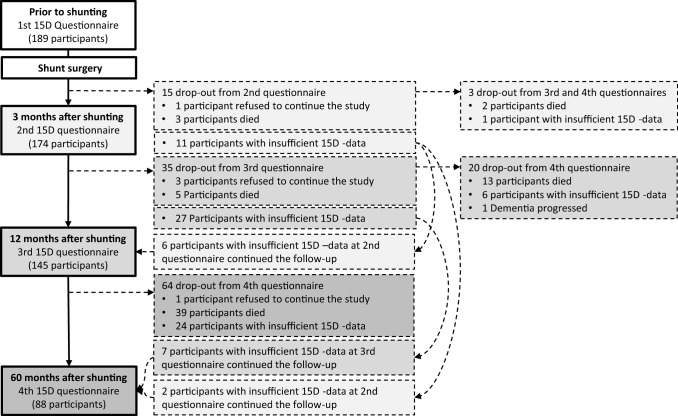
Flowchart of the study population. Legend: Insufficient 15D data, 4 ≥ dimensions missing in the 15D-questionnaire or the questionnaire is missing completely

**Fig. 2:**
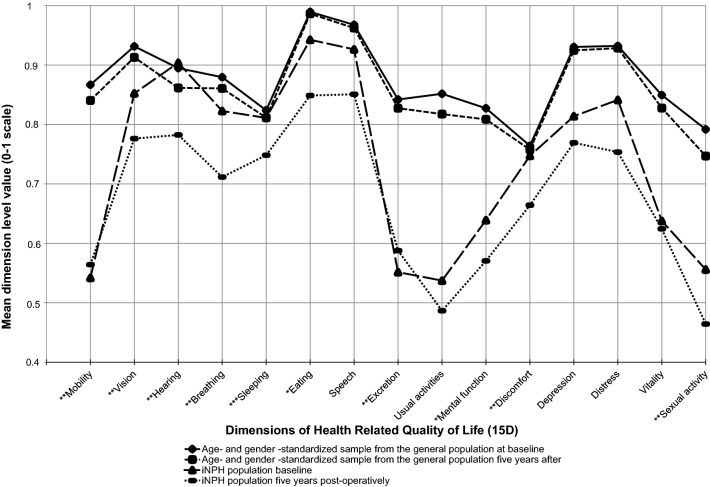
The HRQoL dimensions of the 15D instrument. Comparison of the baseline with 5-year follow-up. Legend: [Number of observations], *denotes significant change in the Wilcoxon Sign test from the baseline to the 1-year follow-up at the *p* < 0.05 level, ** at the *p* < 0.01 level, and the *** at the *p* < 0.001 level. *HRQoL* Health-Related Quality of Life

Out of the 189 initially enrolled study participants, 88 (46%) completed the HRQoL follow-up (Table 2), 64 (34%) had died, and 37 (20%) did not complete the HRQoL follow-up, but were alive at the end of the study (Fig. 1, Supplementary Fig. 1). After 5 years, HRQoL of 37/88 participants (42%) remained stable (5/37) or further improved (32/37) (Table 3). After 5 years, the mean 15D score was significantly lower than at baseline and in three- and twelve-month time points (Table 3). Similarly, after 60 months the mean INPHGS total score had returned to the baseline level, decreasing significantly after the initial post-operative follow-up period (Table 3). The post-operative HRQoL decreased at an average rate of − 2.1% per year. From the 15 health dimensions of the 15D, two (mobility and excretion) remained better than at baseline, but a majority of the dimensions deteriorated below the baseline level at 5 years (Fig. 2). The mean 15D score of the iNPH cohort 5 years after the shunting (0.676, SD 0.16, *n* = 88) was significantly lower as compared with an age- and gender-standardized sample of the general population (0.857, SD 0.04, *n* = 2906, independent samples *t* test *p* < 0.001).

**Table 2:** Characteristics and comorbidities of the study population

	Observed (SD or %)	Number of observations
Age (at shunting)	72 (7.7 ±)	88
Gender (female)	40 (46%)	88
Body mass index (at shunting)	27 (4.8 ±)	85
Education level (9 ≤ years of education)	51 (58%)	84
Severity of depressive symptoms (BDI-21 score, 0–63 scale)	11 (7.3 ±)	52
Cognition level (MMSE score, 0–30 scale)	23 (4.4 ±)	85
Comorbidity burden (median Age Adjusted Charlson Comorbidity Index score)	5 (4,6)^a^	88
Histology in frontal cortical biopsy		88
No Alzheimer’s disease related pathology	49 (56%)	
Amyloid beta	31 (35%)	
Amyloid beta and hyperphosphorylated tau	8 (9%)	
Prognostic tests used prior shunting		88
Tap test	36 (41%)	
Tap and infusion tests	21 (24%)	
Tap and infusion tests and intracranial pressure monitoring	6 (7%)	
Intracranial pressure monitoring	24 (28%)	

*MMSE* mini-mental state examination, *BDI-21* 21-Item Beck Depression Inventory

^a^25th and 75th percentile

**Table 3:** Follow-up of the 88 study participants

	Follow-up								Comparisons		
	Baseline	SD or %	3 months	SD or %	12 months	SD or %	60 months	SD or %	*p* Baseline versus 60 months	*p* 3 months versus 60 months	*p* 12 months versus 60 months
Health-related quality of life (15D score, 0–1 scale)	**0.731**	*0.10* ±	**0.752**^** g**^	*0.12* ±	**0.741**^**f**^	*0.12* ±	**0.676**	*0.16* ±	**0.002**^d^	** < 0.001**^**e**^** (-4.2)**^**j**^	** < 0.001**^**e**^** (4.2)**^**j**^
Health related quality of life improves or remains unchanged at baseline^a^							**37**	*42%*			
Severity of iNPH symptoms (INPHGS score, 0–12 scale)	**5.4**	*2.6* ±	**4.8**^**i**^	*2.7* ±	**4.9**^** h**^	*2.8* ±	**6.1**^c^	*2.9* ±	0.115^e^ (1.6)^j^	**0.001**^**e**^** (-3.2)**^**j**^	**0.006**^**e**^** (-2.8)**^**j**^
Clinically significant decrease in iNPH symptoms^b^							**30**	*36%*			
Shunt valve settings adjusted externally during the follow-up							**52**	*59%*			
One adjustment							**28**	*32%*			
Two or more adjustments							**24**	*27%*			
Time to first adjustment (mean, months)							**11**	*16%*			

*iNPH* idiopathic normal pressure hydrocephalus, *iNPHGS* iNPH Grading Scale

^a^Clinically significant improvement or stability of HRQoL (Δ15D score > -0.015)

^b^iNPHGS decreased at least one point

^c^5 study participants had missing 5-year INPHGS data

^d^Paired-samples test

^e^Wilcoxon signed-rank test

^f^9 study participants had missing 1-year 15D data

^g^6 study participants had missing 3 months 15D data

^h^7 study participants had missing 12 months INPHGS data

^i^6 study participants had missing 3 months INPHGS data

^j^Z-score for the Wilcoxon signed-rank test

Multivariate binary logistic regression analysis was performed with a favorable change in the 15D score 5 years after the shunting as the dependent variable (Table 4). According to the model, younger age (adjusted OR 0.86, 95% CI 0.77–0.95; *p* < 0.005), lower BMI (adjusted OR 0.87, 95% CI 0.77–0.98; *p* < 0.05) and better MMSE performance (adjusted OR 1.16, 95% CI 1.01–1.32; *p* < 0.05) before surgery predicted favorable HRQoL outcome in the 5-year follow-up. The model had an acceptable goodness-of-fit as demonstrated by the Hosmer–Lemeshow test (Table 4) and the overall percentage accuracy rate for the model was 72%. The lowest tolerance was 0.50 (age at shunting) and the highest variance inflation factor was 2.0 (age at shunting) suggesting that multicollinearity did not have a significant effect on the model.

**Table 4:** Multivariate logistic regression analysis for the prediction of 5-year health-related quality of life outcome

Predictors	*n*	Unstandardized coefficient B	SE	Wald’s *χ*^2^	*p*	Adjusted OR (95% CI)
Presence of amyloid beta or hyperphosphorylated tau pathology in the frontal cortical biopsy (= 1, otherwise 0)	83	0.277	0.56	0.24	0.623	1.32 (0.44–3.98)
Age (at shunting)	83	− 0.16	0.05	9.23	**0.002**	**0.86 (0.77**–**0.95)**
Age adjusted Charlson Comorbidity Index score	83	0.35	0.19	3.62	0.057	1.42 (0.99–2.05)
Baseline INPHGS score	83	0.06	0.11	0.31	0.580	1.06 (0.86–1.32)
Baseline MMSE score	83	0.15	0.07	4.73	**0.030**	**1.16 (1.01**–**1.32)**
Body mass index	83	− 0.14	0.06	5.14	**0.023**	**0.87 (0.77**–**0.98)**
Gender (1 = male, 0 = female)	83	− 0.35	0.51	0.48	0.490	0.71 (0.26–1.90)
Constant		9.22	4.12	5.00	0.025	10,083.48
Multivariate model evaluation				*χ*2	*p*	
Overall model evaluation				17.19	0.016	
Goodness-of-fit test (Hosmer and Lemeshow)				8.44	0.392	

*MMSE* mini-mental state examination, *OR* odds ratio, *SE* standard error, *CI* confidence interval

At 5 years, 49/189 (26%) patients had experienced at least one shunt-related complication (Supplementary Fig. 1, Supplementary Table 1), shunt infection being the most common complication (11/189, 6%). The average time from surgery to the first complication was 14 months. The setting of programmable shunt valves was adjusted at least in 101/189 (53%) of the patients. In patients needing valve adjustment, 41/101 (41%) were adjusted twice or more. On average, the first adjustment took place 9 months post-operatively. The most common reason for adjustment was persisting or re-emerging iNPH -related symptoms (77/101, 76%) and the rest were done due to overdrainage (headache, slit ventricles, conservatively manageable subdural effusion/hematoma).

In patients who died during the 5-year follow-up (*n* = 64) the most common causes of death were cardiovascular (17/64, 27%), dementia (11/64, 17%) and cerebrovascular (8/64, 13%) (Supplementary Table 2). The mean time from surgery to death was 32.9 months (SD 18.4).

A mortality analysis was performed using multivariate Cox Proportional-Hazards Model (Supplementary Table 3). Proportional hazard assumption was tested, and it was met. According to the model, presence of amyloid beta or hyperphosphorylated tau pathology in frontal cortical biopsy (39% vs. 22%, absolute risk difference, 17%; HR = 1.99, 95% CI 1.07–3.73; *p* < 0.05) and higher ACCI score (HR = 1.18, 95% CI 1.04–1.35; *p* < 0.05) predicted increased mortality. The highest variance inflation factor was 1.6 (age at shunting) and the lowest tolerance was 0.6 (age at shunting) suggesting that multicollinearity did not have a significant effect on the model.

Post-operative factors (valve adjustments and complications) did not affect the HRQoL outcome in tertiary logistic regression analysis.

## Discussion

### Interpretation

This prospective cohort study was conducted to follow HRQoL of iNPH patients for 60 months after shunt surgery to characterize factors affecting outcome. The extended follow-up showed that the self-evaluated HRQoL outcome is better in iNPH patients with younger age, normal bodyweight and better cognitive status.

During the initial follow-up period of 12 months there was amelioration of iNPH-related symptoms that was reflected in the improvement of HRQoL. However, after 5 years, we observed that this initial post-operative improvement had disappeared. We believe that this may reflect the natural progression of iNPH, but some of this change is more likely due to the aging and comorbidities of the cohort. This is evident when individual HRQoL dimensions are investigated: the observed self-evaluated worsening of breathing or vision are not part of the iNPH symptomology [19]. Because older age is associated with lower HRQoL in the general population (Fig. 2) [12], it is not reasonable to assume that HRQoL would remain the same for 5 years even in persons without iNPH. Therefore, we redefined our original definition for favorable HRQoL outcome to encompass those iNPH patients whose HRQoL remained the same, i.e., 42% of the patients in our cohort. In iNPH patients the HRQoL dimensions of mobility and excretion functions remained above baseline 5 years after shunt surgery, but the mean HRQoL was lower in comparison with the age and gender-standardized general population. In this cohort, the long-term outcome could be considered grim: after 5 years 115/152 persons were either dead or worse off in terms of HRQoL than their pre-operative baseline. However, it must be emphasized, that there is just too little we know about the potential future of patients who are left untreated. Since long-term HRQoL follow-up data from untreated iNPH patients (natural course of the disease) do not exist, one can only assume that it would be lower than in pre-operative situation of the current study population [19–21].

According to the results, a 5-year postoperative, self-evaluated HRQoL was predicted by the persons’ age, cognitive function and BMI before the surgery. While these factors may seem self-evident at first sight, the literature suggests otherwise. Patients with cognitive impairment or with Alzheimer’s disease usually rate their self-rated HRQoL higher than their proxies [22, 23]. We believe this to be the case also in iNPH; however, in what degree, remains to be answered. In iNPH as well as in other forms of dementia, the severity of neuropsychiatric symptoms (such as depression or apathy) heavily impair patients’ HRQoL [2, 22]. Obesity has been associated with lower HRQoL [24], but in the context of iNPH, there is more to be considered. While a recent retrospective study [2] did not find a connection between obesity (BMI ≥ 30 or abdominal obesity measured by waist-to-hip ratio) and HRQoL, it found that hyperlipidemia was associated with lower HRQoL. The authors postulated that the reason for this was comorbid vascular disease [2]. However, as these findings are based on a retrospective cohort, they should be interpreted with appropriate caution. We were unable to reproduce the results of two previous studies reporting an effect of gender on HRQoL [3, 7].

When making conclusions from this cohort, one must take into consideration the subgroup of 37 patients who dropped out but who were alive at the end of the study. There was certainly some whose declining cognitive state was the main reason for the drop out. However, we did not find statistically significant tendency that would skew the results and lead to overestimation. Supplementary proxy-rated HRQoL measure would have been beneficial especially in this subgroup (see the “Limitations and generalisability” section). More research is warranted.

Justified by the significant dropout caused by death, an in-cohort mortality analysis was performed. There were proportionally more deaths in those presenting Alzheimer’s disease (AD)-related pathology and in those with an increased comorbidity burden. While the comorbidity burden in our study is in line with a recent Swedish study [18], it is notable that even in Finnish cohorts with similar follow-up periods, AD-related pathology has not always been associated with increased mortality [25]. This difference might be due to the used model, as the previous study did not take into account the comorbidity burden in their model [25]. At first, we believed that the reason why the comorbidity burden or the cortical biopsy findings [4] failed to predict the 5-year HRQoL outcome was caused by the increased mortality (drop-out) in these groups. To test this hypothesis, we performed an artificial regression model where the deceased were coded to have a HRQoL score of zero after death. Despite this, the comorbidity burden or the cortical biopsy findings did not reach statistical significance. Replication of these results in other cohorts is warranted.

Our results indicate that there is a significant risk of non-lethal complications in long-term follow-up. In this study, we took a broader perspective to post-operative complications, including also falls as a category to provide more comprehensive real-life picture of the risks involved. Surprisingly, the non-lethal complications did not affect long-term HRQoL outcome in our cohort. We do believe that the HRQoL temporarily decreases after non-lethal complication, as shown by a previous study [5], but a swift clinical response can prevent further problems and restore the long-term HRQoL.

An additional caveat of this study is the detailed history of shunt adjustments. In clinical practice, there are individual patients that are in a vicious cycle of readjustments [26] that we believe to decrease the individual HRQoL. In this study, we did not observe worse long-term HRQoL outcomes in patients who required more shunt adjustments. However, in line with the non-lethal complications, these patients may experience a temporary dip in HRQoL.

A number of important clinical implications arise from the current findings. Firstly, HRQoL and severity of iNPH-related symptoms continued to worsen from 12 to 60 months, with a similar rate as they do from 3 to 12 months. Thus, a stable situation at 12 months is not an indication for no further risk of deterioration. If iNPH-related symptoms re-emerge later, some patients may experience an alleviation of symptoms by careful stepwise adjustment of valve setting [26]. An annual follow-up in a neurological unit or health center with a possibility of neurosurgical referral would be able to detect this progression. Lack of worsening at 12 months should not be an indication for neglecting further follow-up.

## Limitations and generalisability

The study did not find any specific parameters that would predict an unfavorable HRQoL outcome. Neither can it be answered whether a specific patient should undergo CSF shunting. In addition to those parameters evaluated at 12-month follow-up [4], proxy-rated HRQoL measure, long-term follow-up of cognitive impairment, depressive symptoms and performance of activities of daily living would have strengthened the study. Self-assessment on the background of cognitive impairment should always be interpreted with reasonable caution. The samples from the general population were cross-sectional. A prospective 15D follow-up of the general population would have given a more detailed view of the impact of age on HRQoL, unfortunately to our knowledge, such a study has not been performed.

## Supplementary Information

Below is the link to the electronic supplementary material.

Supplementary file1 (DOCX 16 KB)

Supplementary file2 (DOCX 15 KB)

Supplementary file3 (DOCX 16 KB)

Supplementary file4 (PDF 1206 KB)
