# Supplementary material for: 5-Year health-related quality of life outcome in patients with idiopathic normal pressure hydrocephalus
Source: J Neurol. 2021 Mar 2;268(9):3283–93. doi: 10.1007/s00415-021-10477-x (PMC8357651; doi:10.1007/s00415-021-10477-x)
Supplement: Supplementary file 1 — Supplementary file1 (DOCX 16 KB) [file 415_2021_10477_MOESM1_ESM.docx]

Supplementary Table 1. Complications of study participants

| **COMPLICATIONS** | Total cohort | 88 patients who completed the 5-year health-related quality of life follow-up |
| --- | --- | --- |
| **Patients that had at least one complication in the 5-year follow-up** | 49 patients (49/189, 25.9%) | 16 patients (16/88, 18.2%) |
| One complication | 36 patients (36/189, 19%) | 12 patients (12/88, 13.6%) |
| Two or more complications | 13 patients (13/189, 6.9%) | 4 patients (4/88, 4.5%) |
| Time to first complication (mean, SD) | 14 months (16.2±) | 17 months (18.8±) |
| **SUM OF ALL OBSERVED COMPLICATIONS** | 70 (49 persons) | 24 (16 persons) |
| **Shunt infection** | 11 (11 persons, 11/189, 5.8%) | 4 (4 persons, 4/88, 4.5%) |
| **Surgical revision** | 43 (30 persons, 30/189, 15.8%) | 15 (11 persons, 11/88, 12.5%) |
| 19 Distal catheter migration (16 into the abdominal wall, 3 perforating the skin) |  |  |
| 7 Overdrainage resulting to chronic subdural hematoma that required trepanation |  |  |
| 11 Shunt valve malfunction |  |  |
| 2 Proximal catheter hemorrhage requiring revision |  |  |
| 2 Distal catheter suture rupture requiring revision |  |  |
| 1 Distal catheter hemorrhage requiring revision |  |  |
| **Perioperative complications** | 5 (5 persons, 5/189, 2.6%) | 1 (1 person, 1/88, 1.1%) |
| 1 Fatal intraventricular hemorrhage |  |  |
| 2 Status epilepticus |  |  |
| 1 Ischemic stroke |  |  |
| **Fall** | 11 (11 persons, 11/189, 5.8%) | 4 (4 persons, 4/88, 4.5%) |
| 4 Acute subdural hematoma |  |  |
| 7 Chronic subdural hematoma |  |  |
