# Supplementary material for: 5-Year health-related quality of life outcome in patients with idiopathic normal pressure hydrocephalus
Source: J Neurol. 2021 Mar 2;268(9):3283–93. doi: 10.1007/s00415-021-10477-x (PMC8357651; doi:10.1007/s00415-021-10477-x)
Supplement: Supplementary file 2 — Supplementary file2 (DOCX 15 KB) [file 415_2021_10477_MOESM2_ESM.docx]

Supplementary Table 2. Causes of death of 64 study participants

| **Classifications of primary cause of death (ICD-10)** | **Number of subjects** | **% of total causes of deaths** |
| --- | --- | --- |
| Diseases of the circulatory system (I, excl. I60-I69) | 17 | 26.6 |
| Dementias (F00-F03, G30) | 11 | 17.2 |
| Cerebrovascular diseases (I60-I69) | 8 | 12.5 |
| External causes (V,W, S, X, Y) | 7 | 10.9 |
| Neoplasms and diseases of the blood (C-D) | 7 | 10.9 |
| Hydrocephalus (G91) | 6 | 9.4 |
| Diseases of the nervous system (G excl. G30, G91) | 3 | 4.7 |
| Diseases of the respiratory system (J excl. J00-J22) | 2 | 3.1 |
| Endocrine, nutritional or metabolic diseases (E) | 1 | 1.6 |
| Infectious diseases (A-B, J00-J22, N39) | 1 | 1.6 |
| Other F. excl. F00-F03, L, M, N, excl. N39, Q) | 1 | 1.6 |

*ABBREVIATIONS: ICD-10, International Statistical Classification of Diseases and Related Health Problems 10^th^ revision.*
