# Supplementary material for: 5-Year health-related quality of life outcome in patients with idiopathic normal pressure hydrocephalus
Source: J Neurol. 2021 Mar 2;268(9):3283–93. doi: 10.1007/s00415-021-10477-x (PMC8357651; doi:10.1007/s00415-021-10477-x)
Supplement: Supplementary file 3 — Supplementary file3 (DOCX 16 KB) [file 415_2021_10477_MOESM3_ESM.docx]

Supplementary Table 3. Cox Proportional-Hazards Model

| **Predictors** | n | Unstandardized  Coefficient B | SE | Wald’s χ^2^ | P | Adjusted  Hazard ratio (95% CI) |
| --- | --- | --- | --- | --- | --- | --- |
| Presence of Amyloid beta or hyperphosphorylated tau pathology in the frontal cortical biopsy (= 1, otherwise 0) | 173 | 0.69 | 0.32 | 4.66 | **0.031** | **1.99 (1.07–3.73)** |
| Age (at shunting) | 173 | -0.04 | 0.03 | 0.02 | 0.899 | 1.00 (0.94–1.05) |
| Age Adjusted Charlson Comorbidity Index score | 173 | 0.17 | 0.07 | 6.12 | **0.013** | **1.18 (1.04–1.35)** |
| Body mass index | 173 | 0.02 | 0.03 | 0.01 | 0.942 | 1.00 (0.94–1.06) |
| Gender (1=male, 0=female) | 173 | -0.30 | 0.28 | 1.19 | 0.276 | 0.74 (0.43–1.27) |
|  |  |  |  | χ2 | P |  |
| Overall model evaluation |  |  |  | 16.39 | 0.006 |  |
| Goodness-of-fit test (Hosmer and Lemeshow) |  |  |  | 10.38 | 0.239 |  |

*LEGEND:* Abbreviations: SE,standard error; CI, confidence interval.
