# Supplementary figures and images for: 5-Year health-related quality of life outcome in patients with idiopathic normal pressure hydrocephalus
Source: J Neurol. 2021 Mar 2;268(9):3283–93. doi: 10.1007/s00415-021-10477-x (PMC8357651; doi:10.1007/s00415-021-10477-x)

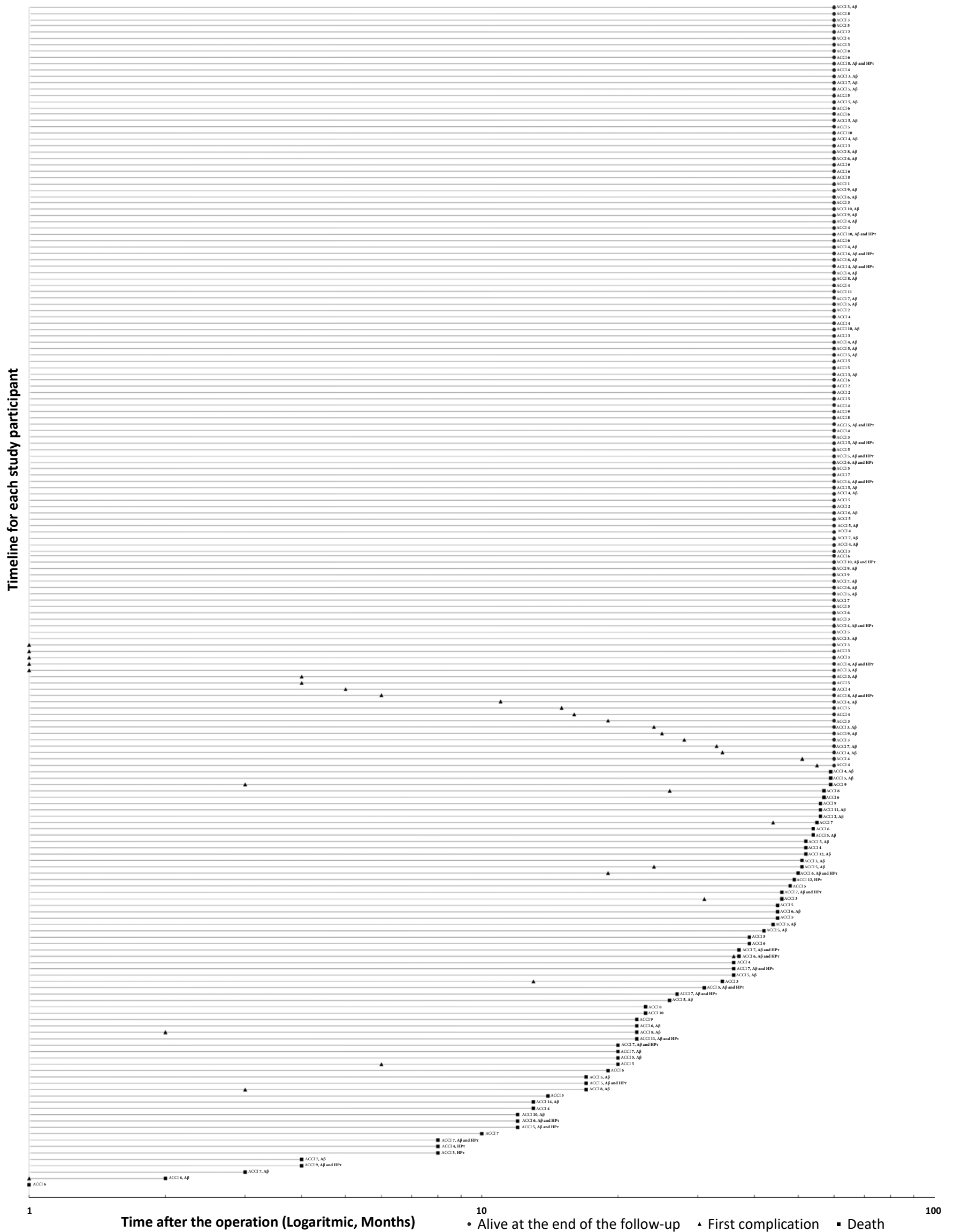

Supplement: Supplementary file 4 — Supplementary file4 (PDF 1206 KB) [file 415_2021_10477_MOESM4_ESM.pdf]
